# Preoperative Prediction of Cervical Lymph Node Metastasis Using Primary Tumor SUVmax on ^18^F-FDG PET/CT in Patients with Papillary Thyroid Carcinoma

**DOI:** 10.1371/journal.pone.0144152

**Published:** 2015-12-04

**Authors:** Ji-hoon Jung, Choon-Young Kim, Seung Hyun Son, Do-Hoon Kim, Shin Young Jeong, Sang-Woo Lee, Jaetae Lee, Byeong-Cheol Ahn

**Affiliations:** Department of Nuclear Medicine, Kyungpook National University School of Medicine and Hospital, Daegu, Republic of Korea; Penn State Hershey Cancer Institute, UNITED STATES

## Abstract

**Objectives:**

The aim of the current study was to evaluate the value of preoperative ^18^F-FDG (FDG) PET/CT in predicting cervical lymph node (LN) metastasis in patients with papillary thyroid carcinoma (PTC).

**Methods:**

One hundred and ninety-three newly diagnosed PTC patients (M: F = 25:168, age = 46.8 ± 12.2) who had undergone pretreatment FDG PET/CT and had neck node dissection were included in this study. The FDG avidity of the primary tumor and the SUVmax of the primary tumor (pSUVmax) were analyzed for prediction of LN metastasis. Detectability by ultrasonography (US) and FDG PET/CT for cervical LN metastasis were also assessed and compared with the pSUVmax.

**Results:**

The FDG avidity of the primary tumor was identified in 118 patients (FDG avid group: 61.0%, M: F = 16:102, age 47.0 ± 12.7 years) and pSUVmax ranged from 1.3 to 35.6 (median 4.6) in the FDG avid group. The tumor size in the FDG avid group was bigger and there was a higher incidence of LN metastasis compared to the FDG non-avid group (0.93 vs. 0.59 cm, p <0.001 and 49.2 vs. 33.3%, p <0.05). In the FDG avid group, patients with LN metastasis had higher pSUVmax than patients without LN metastasis (8.7 ± 8.3 vs. 5.7 ± 5.1, p <0.001). The incidence of central LN metastasis in patients with a pSUVmax >4.6 was 54%; however, the detectability of central LN metastasis by US and FDG PET/CT were 10.3% and 3.6%, respectively.

**Conclusion:**

A high FDG avidity of the primary tumor was related to LN metastasis in PTC patients. Therefore, patients with a high pSUVmax should be cautiously assessed for LN metastasis and might need a more comprehensive surgical approach.

## Introduction

Papillary thyroid carcinoma (PTC) is an endocrine neoplasm with a high incidence of lymphatic metastasis. The incidence of cervical lymph node (LN) metastasis in patients with well-differentiated thyroid carcinoma has been reported to be 20–90% [1]. The metastatic spread of PTC to cervical LN increases the risk of local or regional recurrence of the tumor and the need for further surgery [2, 3]. In addition, some large studies even reported reduced survival because of cervical LN metastases in PTC patients [4, 5]. Since determining the presence of cervical LN metastasis may suggest a different operation method, it is clinically important. Current guidelines recommend removal of all clinically involved metastatic LNs in the central and lateral compartments, which are visualized by preoperative radiological investigations using, for example, ultrasonography (US), enhanced computed tomography (CT), and magnetic resonance imaging (MRI) [6]. However, preoperative CT, MRI, cervical US and ^18^F-fluoro-2-deoxy-D-glucose-positron emission tomography–computerized tomography (FDG PET/CT) has a low sensitivity (30–40%) for detecting cervical LN metastasis [7].

FDG PET/CT is widely used in the staging of various malignancies and is useful in identifying occult LN or distant metastasis [8–14]. Although FDG PET/CT has been used primarily for recurrence work-up in differentiated thyroid carcinoma patients with increased serum thyroglobulin level but negative radioiodine whole body scan, [15, 16] a substantial portion of PTC patients have preoperative FDG PET/CT by incidental first recognition of the PTC on the FDG PET/CT which was performed for other purposes, such as the staging of other malignancies and work-up for fever of unknown origin [17]. However, so far, only a few studies on the value of FDG PET/CT in the preoperative regional LN staging of differentiated thyroid carcinoma have been done. Its role has not yet been fully evaluated [18]. In previous studies, the value of FDG PET/CT for detecting metastatic LN was assessed and the results of the studies precluded the use of FDG PET/CT for detecting metastatic LNs because of a limited ability to detect the metastatic lymph nodes [19, 20]. The limited ability of FDG PET/CT for detecting metastatic LNs warrants other FDG PET/CT approaches to the achievement of better accuracy for predicting LN metastasis. Metabolic parameters of primary tumor on FDG PET/CT has been used to predict occult LN metastasis in various cancers including lung cancer [21], cervical cancer [22], and breast cancer [23]. Even though Byun et al. reported that the highest SUV (SUVmax) are valuable for predicting LN metastasis from papillary thyroid carcinoma, this previous study evaluated only small sized papillary thyroid carcinoma less than 1cm [24]. 45% of enrolled patients had FDG non-avid primary tumor and they assessed the value of FDG PET/CT in the patients with FDG avid primary tumor. Furthermore, they didn’t statistically compared the diagnostic accuracy of SUVmax criterion to that of US. The authors in the current study therefore investigated the value of preoperative FDG PET/CT in predicting cervical LN metastasis in patients with PTC, regardless of tumor size and FDG avidity. Ultimately, we compared the diagnostic accuracy of SUVmax criterion to that of US.

## Materials and Methods

### Patients

In this retrospective study, we analyzed the clinical data of 236 patients with PTC who had preoperative neck US and FDG PET/CT from January 2012 to December 2012. The patients who underwent the surgery in other institutions were excluded because they had no any information and pathologic reports (n = 33). And the patients didn’t received LN dissection were also excluded because the regional LN cannot be assessed (n = 10). Finally, 193 patients (mean age: 46.8 ± 12.2) were enrolled in this study. A hemithyroidectomy with central LN dissection (n = 66) was performed in patients with preoperative cytological diagnoses of PTC but with no evidence of extrathyroid extension or LN metastasis and no suspicious lesions in the contralateral lobes on preoperative US. Otherwise, a total thyroidectomy with LN dissection was performed (n = 127).

### Ethics Statement

The survey was approved by the institutional review board (IRB) of Kyungpook National University School of Medicine/Hospital, Korea. The patient records and information was anonymized and de-identified prior to analysis.

### FDG PET/CT

Patients fasted for at least 6 h and blood glucose levels were checked before the administration of FDG. Patients with elevated blood glucose levels had their examinations rescheduled. None of the patients had blood glucose levels exceeding 150 mg/dL. Approximately 8.1 megabecquerel (MBq) of FDG per kilogram of body weight was injected intravenously and the scans were performed 1 h after FDG injection. FDG PET/CT scans were performed using a Reveal RT-HiREZ 6-slice CT apparatus (CTI Molecular Imaging) and a 16-slice CT Discovery standard test equipment (STE) apparatus (GE Healthcare). For attenuation correction before the PET scan, a low-dose CT scan was obtained without contrast enhancement from the skull base to the upper thigh, with the patient supine and breathing quietly. PET scans with a maximum spatial resolution of 6.5 mm (Reveal PET/CT) and 5.5 mm (Discovery PET/CT) were also obtained from the skull base to the upper thigh at 3 min per bed position. PET images obtained by the Reveal PET/CT and Discovery PET/CT scanners were reconstructed with a 128 × 128 matrix, an ordered-subset expectation maximum iterative reconstruction algorithm (4 iterations, 8 subsets), a Gaussian filter of 5.0 mm, and a slice thickness of either 3.0 mm (Reveal PET/CT) or 3.27 mm (Discovery PET/CT).

### US

Thyroid US was performed with a 5- to 12-MHz linear transducer (HDI 5000; Philips Healthcare, Bothell, WA). The cervical LNs were divided into levels in accordance with the system of the American Joint committee on Cancer [25]. LNs showing any of the following findings on US were considered suggestive of metastatic nodes: a short- to long-axis ratio of greater than 0.5, hypoechogenicity, cystic changes, loss of an echogenic fatty hilum, microcalcification, and abnormal blood flow on Doppler US.

### Image analysis

The FDG PET/CT images and FDG avidity were interpreted by 2 experienced nuclear medicine physicians and a final consensus was reached for all patients. If the primary tumor had focal uptake with underlying diffuse uptake, it was also considered as FDG avidity. Regions of interest were manually placed over the area of maximal activity on slices of the primary tumor lesions in attenuation-corrected images and the pSUVmax within the region of interest over primary lesion was obtained (pSUVmax). The pSUVmax was measured only in the FDG avid group because the exact location of the primary tumor on the PET/CT in the FDG non-avid group, due to the pSUVmax of the FDG non-avid group, was equal to or lower than that of the surrounding normal thyroid tissue.

The pSUVmax was calculated using the following formula:

$$\text{pSUVmax}=\frac{\text{maximum activity in region of interest}\;(\text{MBq}/\text{g})}{\left[\text{injected dose}\;(\text{MBq})/\text{body weight}\;(\text{g})\right]}$$

### Statistical analysis

The positive and negative predictive values of US and FDG PET/CT for detecting cervical LN metastasis were calculated, as well as the sensitivity, specificity, and diagnostic accuracy for predicting the presence of the metastasis on a level-by-level analysis. Surgical pathologic results were used as reference standards. A chi-squared (χ2) test and comparison of proportions were used to compare the sensitivity and specificity of US and FDG PET/CT for predicting LN metastasis. An independent *t* test was applied to compare the differences in the degrees of FDG uptake (pSUVmax) in accordance with pathologic parameters such as tumor size, multiplicity, extrathyroid extension, and LN metastasis. Fisher’s exact test was applied to determined risk factors of LN metastasis. Receiver-operating characteristic (ROC) curves for risk factors were then analyzed to predict LN metastasis. To assess the predictive role of pSUVmax for LN metastasis, Fisher’s exact test and ROC analyses were performed. To minimize the effect of tumor size, we made several subgroups according to primary tumor size and then McNemar test was performed. Finally, authors determined the optimal criterion for the prediction of central LN metastasis and made a comparison with the sensitivity of US, using comparison of proportions. Medcalc version 15.4 (Medcalc Software) was used for all analyses. All P values were two sided and statistical significance was accepted for P values of <0.05.

## Results

### Patients’ characteristics

Table 1 shows the characteristics of 193 subjects. 61.1% (n = 118) of the patients showed FDG avidity of primary tumor on preoperative FDG PET/CT and the SUVmax of the tumor with FDG avidity was 7.2 ± 7.0. Pathologic tumor size was 0.8 ± 0.5 cm (range: 0.2–3.9cm), microcarcinoma was seen in 78.2% (n = 151) of the patients and 43.0% (n = 83) had LN metastasis.

**Table 1:** Characteristics of 193 subjects enrolled in this study.

Characteristics		Value
Mean age (year)		46.8 ± 12.2
Gender		
Female		168 (87.0%)
Male		25 (13.0%)
Pathologic characteristics		
Operation type		
Total thyroidectomy		127 (65.8%)
Hemithyroidectomy		66 (34.2%)
Primary tumor size (cm)		0.8 ± 0.5
Microcarcinoma		151 (78.2%)
TNM stage*		
T stage	T1	87 (45.1%)
	T2	0
	T3	84 (43.5%)
	T4	22 (11.4%)
N stage	N0	110 (57.0%)
	N1	83 (43.0%)
Metabolic parameters		
FDG uptake at primary tumor		
FDG avidity		118 (61.1%)
FDG non-avidity		75 (38.9%)
pSUVmax		7.2 ± 7.0

pSUVmax = SUVmax of primary tumor

*Based on TNM (tumor, node, metastasis) staging system defined by the American Joint Committee on Cancer [25]

### Clinical variables associated with central LN metastasis

**Fig 1:**
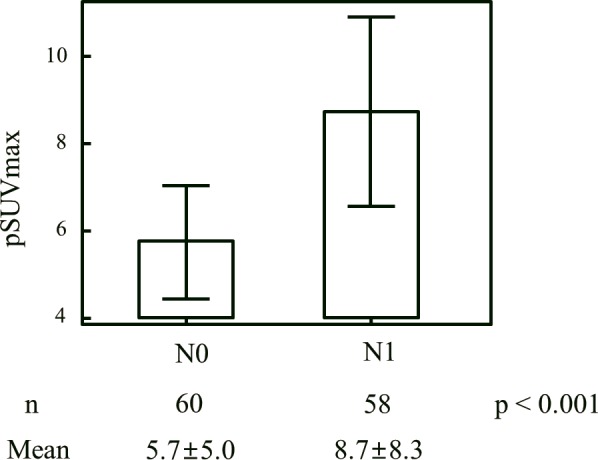
Relationship between SUVmax of primary tumor (pSUVmax) and lymph node metastasis. Fig 1 shows that the pSUVmax was associated with both LN metastasis (p < 0.001) in the FDG avidity group (n = 118).

**Fig 2:**
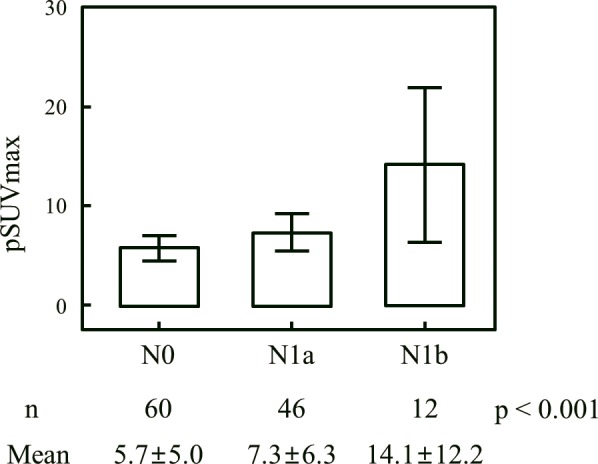
Relationship between pSUVmax and N stage*. Fig 2 shows that the pSUVmax was associated with N stage (p < 0.001) in the FDG avidity group (n = 118).* Based on TNM (tumor, node, metastasis) staging system defined by the American Joint Committee on Cancer.

Table 2 shows the prevalence of LN metastasis according to the presence of FDG avidity at the primary tumor. There was a significant difference in the prevalence of LN metastasis between the FDG avid and FDG non-avid groups. (Figs 1 and 2) shows that the pSUVmax was associated with both LN metastasis (p < 0.001) and N stage (p < 0.001) in the FDG avidity group (n = 118).

**Table 2:** Characteristics of patients according to FDG avidity.

Characteristics	FDG non-avid group	FDG avid group	p value
Mean age (year)	46.5 ± 11.3	47.0 ± 12.7	0.76
Gender			
Female	66	102	0.93
Male	9	16	
Pathologic characteristics			
Primary tumor size (cm)	0.59 ± 0.51	0.93 ± 0.50	<0.001
T stage*			
T1/T2	49	38	<0.001
T3/T4	26	80	
N stage*			
N0	50	60	<0.05
N1	25	58	

* Based on TNM (tumor, node, metastasis) staging system defined by the American Joint Committee on Cancer [25].

### Clinicopathologic variables according to FDG avidity

Table 2 shows the characteristics of patients according to FDG avidity at the primary tumor. The tumor sizes of the FDG non-avidity group were significantly smaller than those of the FDG avid group (0.59 vs. 0.93 cm, p <0.001). There was also a significant difference in the prevalence of LN metastasis between the FDG avid and FDG non-avid groups (49.2 vs. 33.3%, p < 0.05).

### US and FDG PET for detecting LN metastasis

Among 83 patients with LN metastasis, 68 had the metastasis only at the central compartment, 4 had the metastasis only at the lateral compartment, and 11 had the metastasis at both compartments. The sensitivity, specificity, positive predictive value (PPV), and negative predictive value (NPV) of FDG PET/CT for LN metastasis were 3.6, 94.6, 33.3, and 56.5%, respectively. Those for US were 25.3, 92.7, 72.4, and 62.2%, respectively. The sensitivity, specificity, PPV, and NPV for FDG PET/CT for LN metastasis of the central compartment were 2.9, 100, 100, and 62.5%, respectively. Those for US were 10.3, 96.4, 63.6, and 63.5%, respectively.

### Prediction of central LN metastasis

**Fig 3:**
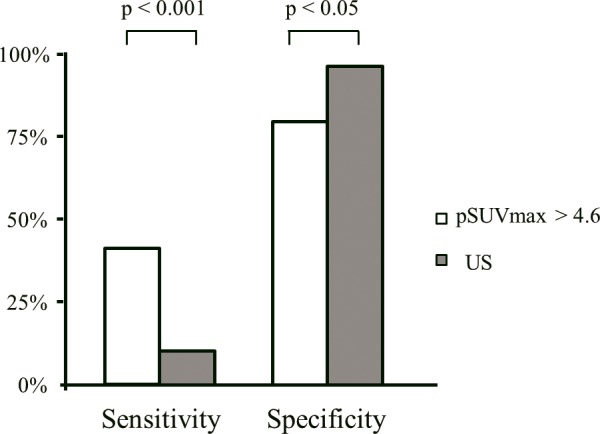
Predictability of central lymph node metastasis by SUVmax of primary tumor (pSUVmax) and ultrasonography (US). Compared to US, the pSUVmax criterion had a significantly higher sensitivity (p <0.001) for predicting central LN metastasis, but a lower specificity (p < 0.05).

The optimal cutoff of pSUVmax for predicting central LN metastasis was 4.6 by ROC analysis and the sensitivity, specificity, PPV, and NPV of the pSUVmax cutoff for predicting central LN metastasis were 58.7, 61.7, 53.6, and 66.4%, respectively. Patients with a pSUVmax >4.6 revealed a higher incidence of central LN metastasis compared to patients with a pSUVmax ≤4.6 (54% vs. 32%, p < 0.05). Compared to US, the pSUVmax criterion had a significantly higher sensitivity (p <0.001) for predicting central LN metastasis, but a lower specificity (p < 0.05) (Fig 3).

### Subgroup analysis for size correction

Of these patients, we made several subgroups according to primary tumor size; group A (tumor size≤1.0, n = 151), group B (tumor size≤2.0, n = 185) and group C (tumor size>2.0, n = 8). In these group A and B, there were significant differences in the prevalence of LN metastasis between the FDG avid and FDG non-avid groups (p < 0.005 and p = 0.0001, respectively), and between the patients with a pSUVmax >4.6 and patients with a pSUVmax ≤4.6 (p < 0.005 and p < 0.005, respectively). While, there was no significant difference in the prevalence of LN metastasis between two groups separated by FDG avid and pSUVmax in group C (p = 1.0 and p = 1.0).

## Discussion

LN metastasis is quite common in patients with PTC and the incidence of cervical LN metastasis has been reported as up to 90% at initial diagnosis [1, 26]. Several studies reported that cervical LN metastasis has prognostic importance for recurrence and survival of PTC [2–5]. In addition to excision of the primary tumor with surrounding normal thyroid tissue, a complete removal of LN metastasis following accurate diagnosis is essential for the cure of PTC. The complete removal of primary and metastatic lesions reduces the recurrence rate. The general agreement on guidelines from various associations about central node dissection (CND) is to dissect central nodes only in cases of confirmed LN metastasis preoperatively or in T3/T4 tumors. However, the role of prophylactic CND remains controversial [27, 28]. Accurate assessment of the cervical LN status is essential for risk stratification and establishment of a treatment plan.

US, the most common imaging modality for preoperative LN staging in patients with thyroid cancer, revealed a relatively high specificity (approaching 95%); however, its sensitivity was quite low (as low as approximately 25%) [20, 29]. It is well known that the detection of metastasis on US is heavily dependent on careful searching by radiologists. In the current study, the sensitivity of US for detecting cervical LN metastasis was lower than in previous reports. This might be partially related to the characteristics of the patients in the current study, given that 78% of the participants had a microcarcinoma. The early thyroid cancer might produce a small metastatic lesion, which is undetectable and incapable of changing the anatomical features of the cervical LN; therefore, the metastatic LN might not be easily detected on US. In addition, the low sensitivity may be related to the surgical protocol of our institution which includes a routine central neck dissection. This subsequently decreases motivation for paying close attention to the central neck node.

FDG PET/CT has been reported to be efficient in staging, restaging, and prognosticating various malignancies, including lung, breast, head, and neck cancers [11, 30]. Although there are published reports [19, 31] about the effectiveness of preoperative FDG PET/CT evaluation for thyroid cancer, it has not been widely used for preoperative staging in patients with differentiated thyroid cancer. This is presumably because of the excellent prognosis of the disease and the low diagnostic sensitivity of FDG PET/CT for the disease. Because of the high incidence of dormant thyroid cancer [32–35], it is one of the common incidentally-found malignancies in the FDG PET/CT study, which was performed for the staging of other malignancies or cancer screening. Therefore, some of the thyroid cancer patients have preoperative FDG PET/CT. The current study aimed to assess the clinical value of preoperative FDG PET/CT in patients with PTC.

Prediction of LN metastasis by pSUVmax had been reported in other cancers [22, 23]. In the patients with gastric cancer, the pSUVmax was an independent indicator of LN metastasis [36]. Using the pSUVmax cutoff value of 3.2, the sensitivity and specificity were 74.6% and 74.0% respectively. Kim et al. reported that a high pSUVmax showed an association with an increased risk of LN metastasis in clinical N0 squamous cell lung carcinoma patients [21]. Using the pSUVmax cutoff value of 8.8, the sensitivity and specificity were 91.7% and 52.9% respectively.

The results of the current study demonstrate that the size of the primary tumor was significantly larger in the FDG avid group than in the FDG non-avid group and that the FDG avidity was significantly associated with LN metastasis. The prevalence of central LN metastasis was higher in PTC patients with pSUVmax >4.6 than in PTC patients with pSUVmax ≤4.6. In addition, a high pSUVmax was associated with the presence of central LN metastasis in patients with microcarcinoma (p < 0.005), even though it was reported that the patients with microcarcinoma had less frequent central LN metastasis [24]. After tumor size correction, group A and B still showed significant correlation between pSUVmax and LN metastasis. In group C, FDG avidity and pSUVmax was not significantly associated with LN metastasis, it might derived from the small number of patients (n = 8), The results of the current study reveal that the pSUVmax might be a better predictor than US for predicting central LN metastasis in patients with PTC. Therefore, central LN dissection might be undertaken in patients with high pSUVmax in order to reduce the chance of residual LN metastasis in the central compartment of the neck.

The study has limitations. Partial volume correction of the SUVmax was not performed in the current study. SUVmax can be underestimated by the partial volume effect, especially in small tumors [37]. Hoetjes et al. reported that correction of the effect may improve the accuracy of the diagnostic performance of SUVmax [38]. Although pSUVmax predicted central LN metastasis successfully in the current study, application of the partial volume correction may improve predictability of pSUVmax for the LN metastasis. Predictability of pSUVmax for lateral LN metastasis, which is more valuable in the clinical field, was not performed in the current study due to inborn referral bias, i.e., lateral neck dissection was performed solely in patients who demonstrated evidence of lateral neck LN metastasis on preoperative radiological evaluation or fine needle aspiration cytology. Lastly, there was relatively wide overlap between patients with LN metastasis and without, therefore, pSUVmax can be used more usefully with other clinical risk factors.

## Conclusion

FDG avidity of primary tumor on preoperative FDG PET/CT can be used to predict LN metastasis with clinical risk factors in patients with PTC. Patients with a high pSUVmax should be cautiously assessed for LN metastasis and might need a more comprehensive surgical approach.
